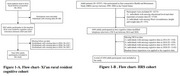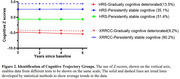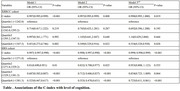# Association Between Conicity Index and Cognitive Trajectories: Evidence From Two Ethnically Distinct Longitudinal Cohorts

**DOI:** 10.1002/alz70860_099059

**Published:** 2025-12-23

**Authors:** Kang Huo, Jin Wang, Qiumin Qu

**Affiliations:** ^1^ The First Affiliated Hospital of Xi’an Jiaotong University, Xi’an, Shaanxi, China; ^2^ The First Affiliated Hospital of Xi'an Jiaotong University, Xi'an, Shaanxi, China

## Abstract

**Background:**

Mild cognitive impairment (MCI) and dementia are global health challenges. Abdominal obesity, measured by the conicity index (CI), has been linked to cognitive impairment, yet longitudinal studies exploring its impact on cognitive trajectories remain limited. This study examines the association between CI and cognitive trajectories in two diverse cohorts and evaluates whether abdominal obesity represents a modifiable risk factor.

**Method:**

This retrospective cohort study utilized data from the Xi’an Rural Resident Cognitive Cohort (XRRCC) and the U.S. Health and Retirement Study (HRS). Participants with normal baseline cognitive function and at least four waves of data (2014–2020) were included. Abdominal obesity was quantified using CI and categorized into quartiles. Latent class growth models (LCGMs) identified cognitive trajectories, and multinomial logistic regression analyzed the association between CI quartiles and trajectories, adjusting for demographic, lifestyle, and clinical variables. Subgroup analyses assessed variations by age, sex, and comorbidities.

**Result:**

In the XRRCC cohort (*n* = 1,452), two cognitive trajectories were identified: persistently stable (90.2%) and gradually deteriorating (9.2%). In the HRS cohort (*n* = 4,494), three trajectories were observed: persistently high (35.1%), persistently moderate (51.4%), and gradually deteriorating (13.5%). Higher CI values were associated with worse cognitive trajectories in both cohorts. Fully adjusted models showed that participants in the highest CI quartile had greater odds of cognitive decline compared to the lowest quartile (XRRCC: OR = 0.554, 95% CI: 0.328–0.938; HRS: OR = 0.725, 95% CI: 0.611–0.861). Subgroup analyses revealed stronger associations among women and younger participants (<65 years).

**Conclusion:**

Higher CI values are linked to worse cognitive trajectories, particularly in women and younger individuals. These findings highlight abdominal obesity as a potential modifiable risk factor for cognitive decline and support early interventions targeting central obesity to mitigate cognitive deterioration.